# Pancreatic Tuberculosis Manifesting as Pancreatic Mass: A Case Report

**DOI:** 10.7759/cureus.88521

**Published:** 2025-07-22

**Authors:** Amit Kumar Meena, Yashasvi Agarwal, Nikhil Batra, Santosh Hajare

**Affiliations:** 1 Gastroenterology, Jawaharlal Nehru Medical College, Belagavi, IND; 2 Internal Medicine, Jawaharlal Nehru Medical College, Belagavi, IND

**Keywords:** diagnosis, immunocompetent, pancreatic biopsy, pancreatic malignancy, pancreatic mass, pancreatic tuberculosis

## Abstract

Tuberculosis (TB) continues to be a major global health burden, particularly in low- and middle-income countries, where it contributes significantly to morbidity and mortality. While pulmonary TB is the most common form, extrapulmonary manifestations, including pancreatic TB, are rare and often pose diagnostic challenges. Isolated pancreatic TB in immunocompetent individuals is exceptionally uncommon and is rarely considered in the initial differential diagnosis of a pancreatic mass. We present the case of a 26-year-old immunocompetent woman with a six-month history of persistent epigastric pain, nausea, vomiting, and progressive weight loss. Laboratory investigations revealed normocytic normochromic anemia and mildly elevated serum bilirubin levels. Contrast-enhanced computed tomography (CT) of the abdomen demonstrated pancreatic mass, leading to a provisional diagnosis of pancreatic carcinoma. However, histopathological examination of the lesion revealed features of an acute suppurative process. Further analysis of aspirated cystic fluid using the Cartridge-Based Nucleic Acid Amplification Test (CBNAAT) and Ziehl-Neelsen (ZN) staining confirmed the presence of *Mycobacterium tuberculosis*. The patient was commenced on standard anti-tubercular therapy, which led to marked clinical improvement. On follow-up, the patient’s symptoms had resolved completely, and repeat imaging demonstrated normalization of pancreatic architecture. This case highlights the importance of considering pancreatic TB as a diagnosis, even in immunocompetent individuals, particularly from endemic regions. Limitations of our study include a short follow-up period and unavailability of endoscopic ultrasound (EUS) evaluation.

## Introduction

Infection with *Mycobacterium tuberculosis *(TB) remains a leading cause of mortality from a single infectious agent, affecting an estimated 10.8 million individuals globally. India alone contributes approximately 26% of the global TB burden [1]. While TB predominantly involves the lungs, extrapulmonary manifestations are observed in about 12.5% of cases. Among these, abdominal TB accounts for 11-16% [2,3].

Isolated pancreatic tuberculosis is extremely rare, with a prevalence of 0.2% to 2%, largely due to the pancreas’s intrinsic resistance to infection, attributed to the bactericidal properties of pancreatic enzymes [3]. However, recent years have seen an increase in reported cases of pancreatic TB. This trend may be attributed to advances in imaging techniques and the development of procedures that facilitate sample acquisition from the pancreas. Additionally, rising numbers of immunocompromised individuals, particularly those with human immunodeficiency virus (HIV) infection and advanced malignancies, have contributed to this increase [2].

The diagnosis of pancreatic TB is particularly challenging due to its rarity and the nonspecific nature of its clinical presentation, which often mimics that of pancreatic malignancy, thereby earning the designation of 'Great Mimicker' [2,4]. A diagnostic triad has been proposed, comprising (1) endoscopic ultrasound-guided fine needle aspiration (EUS-FNA) revealing caseating granulomas, (2) a positive cartridge-based nucleic acid amplification test (CBNAAT), and (3) elevated cystic fluid adenosine deaminase (ADA) levels exceeding 35 IU/L [5]. In addition, Sharma et al. have proposed an alternative diagnostic and therapeutic algorithm for pancreatic TB [6].

Early and accurate diagnosis is critical, as it allows for appropriate initiation of anti-tubercular therapy, potentially avoiding unnecessary surgical intervention and reducing healthcare costs. 90% of patients with a pancreatic mass undergo abdominal computed tomography (CT), with few being subjected to additional magnetic resonance imaging (MRI) and EUS. Despite this, 50% of patients require exploratory laparotomy, indicating high initial misdiagnosis, most commonly as pancreatic malignancy [2]. Here, we present the clinical and radiological findings of a 26-year-old immunocompetent woman from a TB-endemic region, with a complete vaccination history, diagnosed with isolated pancreatic tuberculosis.

## Case presentation

A 26-year-old woman, hailing from India, a TB endemic region, presented with six months of epigastric pain, nausea, non-bilious vomiting, and 7 kg weight loss. Family and past history were non-contributory. She had no history of prior abdominal surgeries, endoscopic procedures, or hospital admissions. There was no known history of prolonged antibiotic use, complementary and alternative medicine, chronic illness, immunosuppressive therapy, or any contact with a known TB patient. The patient reported receiving routine childhood immunization in accordance with the Indian Universal Immunization Program (UIP). She was afebrile with stable vital signs. Abdominal examination showed epigastric tenderness, with a normal physical examination.

**Figure 1:**
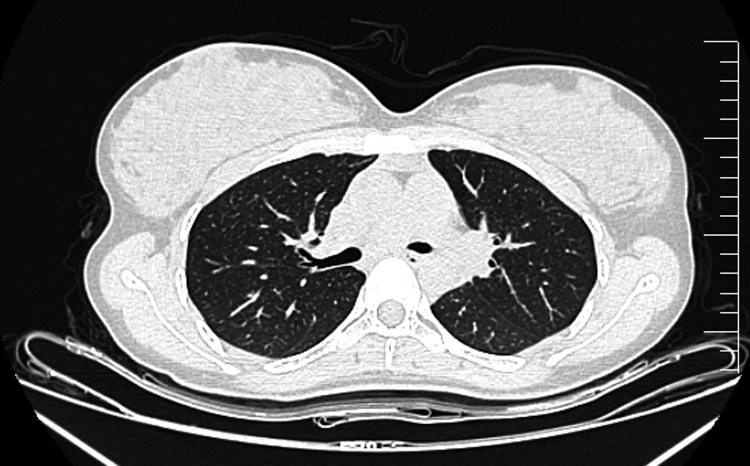
High-resolution computed tomography (CT) scan of bilateral lung fields showing no pathological lesion and no lymphadenopathy.

Laboratory evaluation showed normocytic normochromic anemia (hemoglobin 11.2 mg/dl), reduced vitamin B12 (179 pg/ml), reduced vitamin D (21.4 ng/mL), and mildly elevated total bilirubin (1.31 mg/dl). Other parameters, including erythrocyte sedimentation rate (ESR 16 mm/hr), liver enzymes (alanine aminotransferase 12 IU/l and aspartate aminotransferase 20 IU/l), glycosylated hemoglobin (HbA1C 5.1%), serum amylase (56 IU/l), and serum lipase (26 IU/l), were normal. Carcinoembryonic antigen (CEA) 0.98 ng/ml, carbohydrate antigen 19-9 (CA 19-9) 24.5 IU/ml, and alpha-fetoprotein (AFP) 0.9 ng/ml were normal. Viral markers (hepatitis B, C, and human immunodeficiency virus) and the tuberculin skin test (TST) were negative. Table 1 demonstrates detailed laboratory values. High-resolution chest CT was normal, as demonstrated in Figure 1.

**Table 1:** Laboratory values.

Laboratory Tests	Patient’s Value	Normal Range
Haemoglobin (gm/dL)	11.2	12.0 - 15.0
White Blood Cell Count (cells/mL)	4300	4000 – 10,000
Neutrophils (%)	59	40 – 75
Lymphocytes (%)	32	20 – 42
Platelet Count (cells/mL)	223,000	150,000 – 450,000
Prothrombin Time (PT) (seconds)	13.2	9 – 12.5
International Normalised Ratio (INR)	1.22	0.86 – 1.12
Erythrocyte Sedimentation Rate (ESR) (mm/hr)	16	0 – 20
High sensitive C-Reactive Protein (HsCRP) (mg/L)	0.6	0 – 5.0
Serum Iron (mg/dL)	59	33 – 193
Total Iron Binding Capacity (TIBC) (mg/dL)	253	135 – 392
Transferrin Saturation (%)	19	12 – 45
Serum Vitamin B12 (pg/mL)	179	197 – 771
Serum Vitamin D3 (ng/mL)	21.4	30 – 100
Total Bilirubin (mg/dL)	1.31	0 – 0.9
Direct Bilirubin (mg/dL)	0.5	0 – 0.3
Indirect Bilirubin (mg/dL)	0.81	0 – 1.1
Alanine Aminotransferase (ALT) (U/L)	12	1 – 33
Aspartate Aminotransferase (AST) (U/L)	20	1 – 32
Alkaline Phosphatase (ALP) (U/L)	96	35 – 105
Total Protein (gm/dL)	7.1	6.6 – 8.7
Albumin (gm/dL)	4.4	3.5 – 5.2
Globulin (gm/dL)	2.7	2.0 – 3.9
Random Blood Glucose (mg/dL)	82	70 – 140
Urea (mg/dL)	15.8	16.6 – 48.5
Blood Urea Nitrogen (BUN) (mg/dL)	7.38	8 – 23
Creatinine (mg/dL)	0.5	0.5 – 0.95
Serum Sodium (mmol/L)	136	136 – 145
Serum Potassium (mmol/L)	4.54	3.5 – 5.1
Serum Chloride (mmol/L)	107	98 – 107
Serum Bicarbonate (mmol/L)	17.8	22 – 29
HbA1C (%)	5.1	<5.6
Amylase (U/L)	56	28 – 100
Lipase (U/L)	26	13 – 60
Carcinoembryonic Antigen (CEA) (ng/mL)	0.98	0 – 5.2
Alpha Fetoprotein (AFP) (ng/mL)	0.9	0 – 7
Carbohydrate Antigen 19-9 (CA 19-9) (U/mL)	24.5	0 – 34
Carbohydrate Antigen 125 (CA 125) (U/mL)	12	0 – 35
Total Cholesterol (mg/dL)	101	<200
Triglycerides (TG) (mg/dL)	101	<150
Low Density Lipoprotein (LDL) (mg/dL)	55	<100
High Density Lipoprotein (HDL) (mg/dL)	38	40 – 60
Hepatitis B	Negative	-
Hepatitis C	Negative	-
Human Immunodeficiency Virus (HIV)	Negative	-
Tuberculin Skin Test (TST)	Negative	-

**Figure 2:**
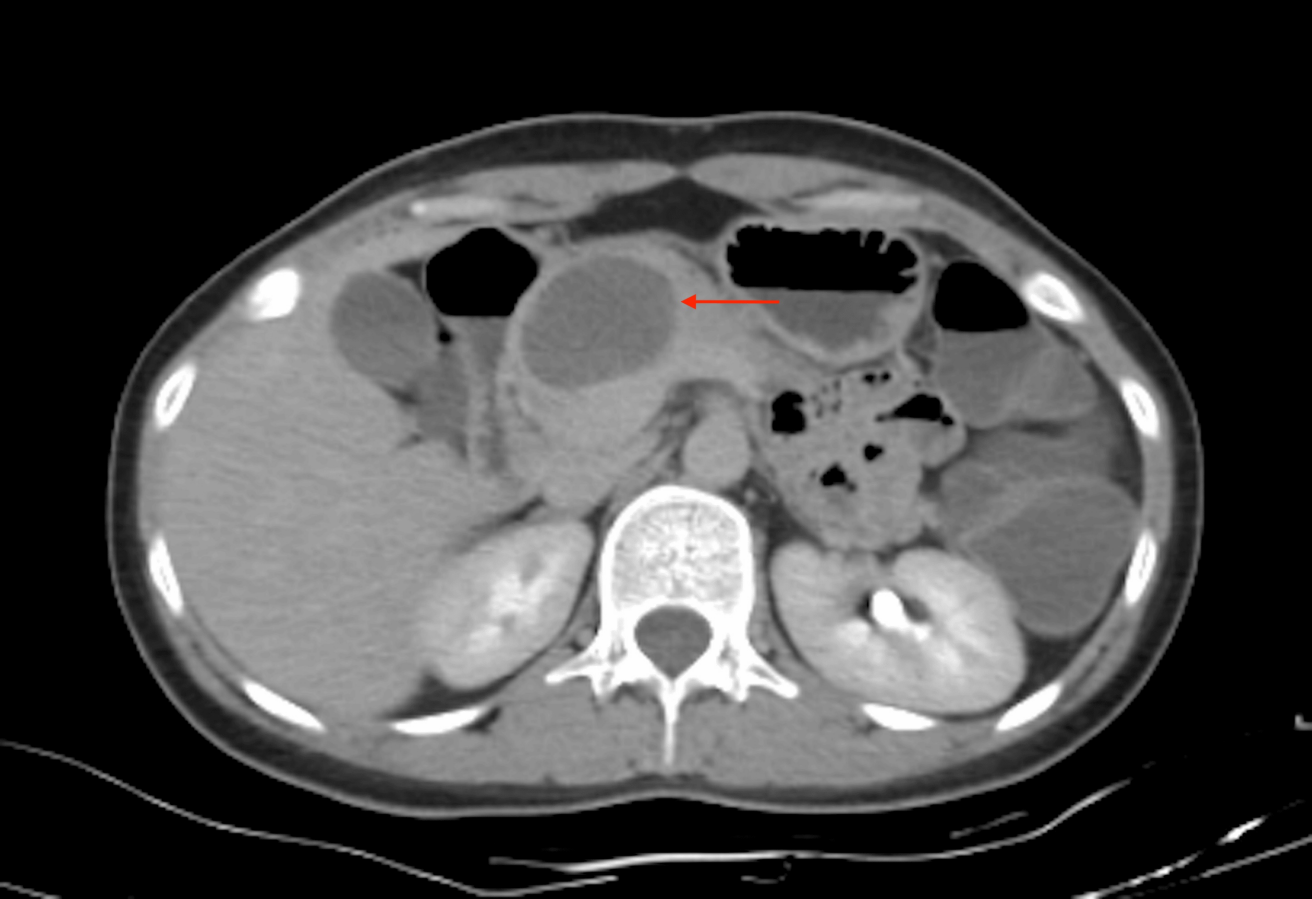
Non-contrast computed tomography (CT) scan of pancreatic mass.

**Figure 3:**
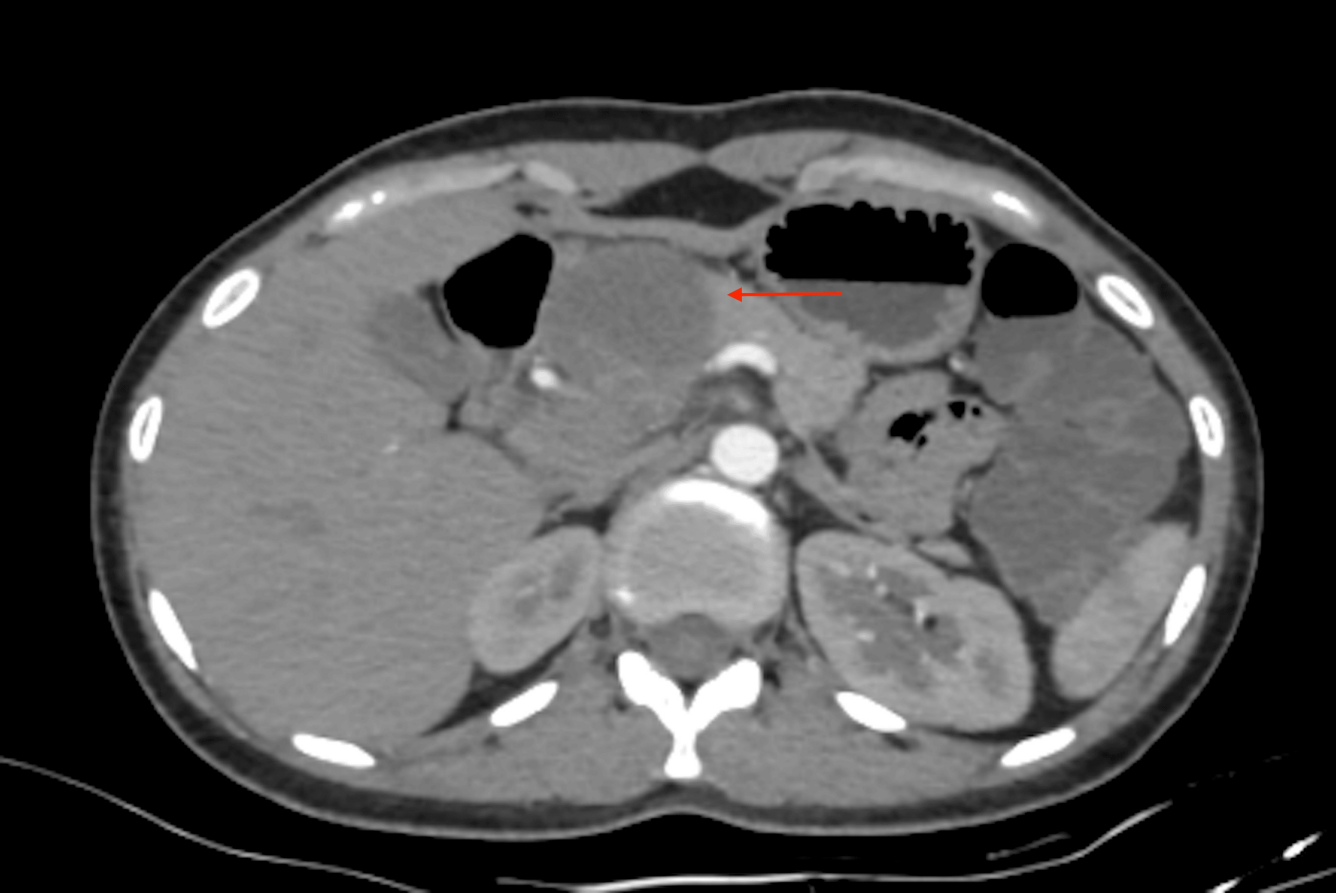
Contrast-enhanced computed tomography (CT) scan of pancreatic mass. There is an irregular, ill-defined, small hypodense area noted in the neck of the pancreas, approximately measuring 2.2x1.8x1.6 cm. No obvious communication with the pancreatic duct. No evidence of calcification or solid component noted. Superiorly, the lesion is seen to extend up to segment three of the left lobe of the liver. Periportal group of lymph nodes noted with few non-enhancing areas, the largest measuring 1.4x0.8 cm. Findings are suggestive of mucinous cystadenoma with necrotic periportal lymphadenopathy.

Abdominal CT showed a 2.2x1.8x1.6 cm thin-walled hypodense cystic lesion in the tail of the pancreas, suggestive of mucinous cystadenoma with necrotic periportal lymphadenopathy, demonstrated in Figures 2, 3.

**Figure 4:**
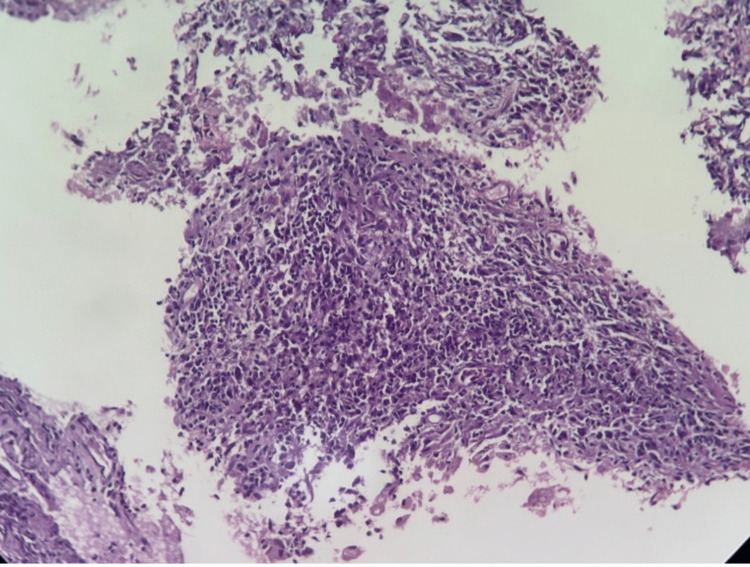
Histopathological examination of pancreatic tissue. It shows neutrophils and macrophages in a necrotic background suggestive of an acute suppurative lesion.

CT-guided biopsy and aspiration were performed. Histopathology demonstrated numerous macrophages and neutrophils in a necrotic background, without granulomas or atypical cells, suggestive of acute suppurative lesion, shown in Figure 4.

Cystic fluid CBNAAT detected TB without rifampicin resistance, and ZN staining was acid-fast bacilli (AFB) positive, with 1+ grading.

A diagnosis of primary pancreatic tuberculosis was made, and the patient was started on weight-based anti-tubercular treatment. She was given tablets of rifampicin 150 mg, isoniazid 75 mg, pyrazinamide 400 mg, ethambutol 275 mg, and pyridoxine 50 mg daily for 2 months, followed by a continuation phase with rifampicin, isoniazid, and ethambutol for four months. Liver enzymes remained stable during follow-up. After treatment, symptoms resolved and a follow-up CT scan revealed lesion resolution. Table 2 provides a detailed summary of the case events.

**Table 2:** Chronological summary of clinical course.

Time Point	Event
Six months prior to admission	Onset of epigastric pain, nausea, non-bilious vomiting, and 7 kg weight loss
Initial presentation	26-year-old woman from India (TB-endemic region) presented with chronic gastrointestinal symptoms
History and examination	No significant family or past medical history. No history of abdominal surgery, endoscopic procedures, or hospital admission. No TB contact. Routine UIP vaccination. Afebrile, stable vitals. Localized epigastric tenderness.
Laboratory investigation (Day 1 of admission)	- Mild normocytic normochromic anemia (Hb 11.2 g/dL) - Low vitamin B12 (179 pg/mL), low vitamin D3 (21.4 ng/mL) - Mildly elevated total bilirubin (1.31 mg/dL) - Normal ESR, CRP, liver enzymes, pancreatic enzymes - Tumor markers (CEA, CA 19-9, AFP) within normal range - Viral markers and Tuberculin Skin Test (TST) negative
Chest Imaging (Day 2 of admission)	High-resolution CT scan: No pathological lesions or lymphadenopathy
Abdominal Imaging (Day 2 of admission)	CT scan showed a 2.2 x 1.8 x 1.6 cm hypodense cystic lesion in the pancreatic tail, with necrotic periportal lymphadenopathy. Suspicious for mucinous cystadenoma
Tissue diagnosis (Day 4 of admission)	CT-guided biopsy and aspiration of pancreatic lesion performed
Microbiological findings (Day 5 of admission)	- CBNAAT of cystic fluid: Positive for Mycobacterium tuberculosis, no rifampicin resistance - Ziehl-Neelsen stain: AFB positive (1+ grading)
Histopathological findings (Day 6 of admission)	Neutrophils and macrophages in necrotic background. No granulomas or atypical cells. Suggestive of acute suppurative lesion
Final diagnosis (Day 6 of admission)	Isolated Pancreatic Tuberculosis
Treatment initiation (Day 6 of admission)	Started on weight-based anti-tubercular therapy (ATT): - Intensive phase (2 months): Rifampicin 150 mg, Isoniazid 75 mg, Pyrazinamide 400 mg, Ethambutol 275 mg, Pyridoxine 50 mg - Continuation phase (4 months): Rifampicin, Isoniazid, Ethambutol
Follow-up (Weekly for 2 weeks, followed by monthly)	Liver, renal functions and complete blood count remained stable
Outcome (After 6 months of treatment initiation)	Complete resolution of symptoms. Follow-up CT showed resolution of the pancreatic lesion.

## Discussion

Pancreatic tuberculosis (TB) is a rare extrapulmonary manifestation, with a prevalence of 0.2% to 2%, even in high TB-burden regions [3]. As per the WHO 2023 report, global TB incidence has risen by 18%, with 32% of pancreatic TB cases reported in immunocompetent individuals [7]. Despite BCG vaccination coverage of 91.44% in India in 2024, TB incidence continues to increase, highlighting the limited efficacy of BCG in preventing adult and extrapulmonary TB forms, such as pancreatic TB [8]. Indian case reports indicate that nearly 50% of pancreatic TB patients are under 30 years of age [2]. We present a case of pancreatic TB in a 26-year-old immunocompetent woman from India, emphasizing the need to consider this diagnosis in young patients from endemic regions.

The pancreas is relatively resistant to tuberculosis, likely due to the enzymatic destruction of *Mycobacterium tuberculosis*. Antibacterial properties of pancreatic secretions, including lipases and deoxyribonucleases, have also demonstrated antimycobacterial effects, contributing to the rarity of pancreatic TB [9]. Pancreatic tuberculosis occurs primarily through hematogenous or lymphatic spread from a distant focus, often the lungs or peripancreatic lymph nodes. It may also result from direct extension of nearby infected structures or reactivation of latent bacilli, particularly in immunocompromised individuals [10]. 

Pancreatic TB lacks pathognomonic clinical or radiological features, making diagnosis difficult. Common symptoms include abdominal pain (74.8%), weight loss (51.6%), and fever (46.5%). Jaundice, diarrhea, acute or chronic pancreatitis, and hemorrhage secondary to splenic vein thrombosis were also reported [2]. It commonly involves the pancreatic head (59%), followed by the body (18.2%), tail (13.4%), and neck (1.8%) [7]. Our patient presented with abdominal pain, vomiting, and noticeable weight loss.

Routine laboratory investigations may reveal anemia, liver function abnormalities, or glucose fluctuations; however, these findings are non-specific and can be seen in a variety of differential diagnoses [11]. Vitamin D3 deficiency also increases the risk for TB susceptibility and disease progression [12]. Amylase and lipase are elevated in only 26.8% and 31.3% of cases, respectively, offering limited diagnostic value [2]. Impairment in exocrine and endocrine function of the pancreas in patients with pancreatic TB remains unexplored [13]. Our patient had normocytic normochromic anemia, mildly elevated bilirubin with low vitamin B12 levels, and vitamin D3 levels.

A Mantoux test is positive in almost 70% of pancreatic TB cases [2], offering a cost-effective, accessible screening tool with early results [8]. Panic et al. recommended TST or interferon-gamma release assay (IGRA) before invasive procedures, especially in low endemic areas [2]. Despite hailing from a TB-endemic area, our patient's TST was negative.

Imaging modalities for pancreatic TB include abdominal ultrasonography (USG), CT, MRI, endoscopic retrograde cholangiopancreatography (ERCP), and EUS [11]. Despite these, over 50% of patients undergo laparotomy due to misdiagnosis [2]. CT is preferred to evaluate pancreatic lesions but cannot reliably distinguish pancreatic TB from carcinoma due to a lack of pathognomonic radiologic features [7].

Studies have compared radiological features to differentiate pancreatic carcinoma from TB. Vascular invasion favors pancreatic carcinoma but can occur in one-third of TB cases [3]. Ductal dilation is common in pancreatic carcinoma but rare in TB. Calcification favors a diagnosis of pancreatic TB [7]. 90% of pancreatic TB cases show necrotic peripancreatic lymph nodes, only seen in 30% of carcinoma cases [5]. Our patient had an ill-defined cystic lesion without vascular invasion, ductal dilation, or calcification.

Clinical and imaging findings alone cannot differentiate pancreatic carcinoma from TB; tissue sampling remains necessary for diagnosis and treatment [14]. EUS is preferred for evaluating suspected pancreatic TB, offering approximately 90% sensitivity and allowing FNA of pancreatic and peripancreatic lymph nodes [11]. When unavailable, CT or USG-guided FNA can also be performed [7]. We performed CT-guided FNA for definitive diagnosis.

Microbiological analysis includes ZN staining, culture, which is not preferred due to the slow growth of the organism, and polymerase chain reaction (PCR), comprising CBNAAT. Histopathology demonstrates caseating granulomas with multinucleated giant cells [4]. Pancreatic mass samples are more diagnostic than lymph nodes, though the ideal FNA site remains unclear [8]. In our patient, histopathological evaluation did not demonstrate classical caseating granulomas typically seen in TB. Instead, a necrotic background with neutrophils and macrophages suggested an acute suppurative lesion. This discrepancy can occur due to sampling errors or partial lesion involvement. Nevertheless, a positive CBNAAT and ZN staining confirmed the diagnosis. The absence of granulomas highlights the importance of using a multimodal diagnostic approach in such cases [4,5].

TB is an infectious disease, with a 94% cure rate. WHO recommends six to twelve months of therapy, depending on response [5]. Treatment effectiveness is monitored by symptom resolution, laboratory improvement, and reduced mass size on CT [4]. Our patient completed six months of anti-tuberculous therapy, which was associated with resolution of the pancreatic mass.

Limitations in our study include non-availability of EUS, the preferred diagnostic modality, and a short six-month follow-up, insufficient to evaluate long-term recurrence and complications. Future studies should include extended follow-up and aim to develop diagnostic and treatment algorithms. Advances in artificial intelligence (AI) offer promising avenues in the early detection and classification of pancreatic lesions. AI-powered radiological tools, such as convolutional neural networks (CNNs), have demonstrated potential in distinguishing between malignant and infectious etiologies on imaging modalities like CT and MRI. Moreover, AI-driven clinical decision support systems can integrate patient data, risk factors, and imaging to prioritize tuberculosis in differential diagnosis, especially in TB-endemic regions. Incorporating AI into diagnostic workflows could reduce misdiagnosis, optimize treatment planning, and improve outcomes in rare conditions like pancreatic TB [15].

## Conclusions

We present the case of a 26-year-old immunocompetent female diagnosed with isolated pancreatic tuberculosis. Although the incidence of pancreatic TB is low, it should be considered in the differential diagnosis of pancreatic masses, particularly in young patients from TB-endemic regions. The absence of standardized guidelines for the diagnosis and management of pancreatic TB, especially in resource-limited settings, represents a significant gap that warrants further investigation. Emerging technologies such as artificial intelligence (AI)-assisted imaging analysis have demonstrated early potential in distinguishing pancreatic TB from pancreatic carcinoma and may aid in improving diagnostic accuracy. Timely recognition of pancreatic TB is crucial, as it allows for appropriate medical management, reduces the risk of unnecessary surgical intervention, and ultimately improves patient outcomes.

## References

[1] (2025). WHO: Global tuberculosis report 2024. https://www.who.int/teams/global-programme-on-tuberculosis-and-lung-health/tb-reports/global-tuberculosis-report-2024.

[2] Panic N, Maetzel H, Bulajic M (2020). Pancreatic tuberculosis: a systematic review of symptoms, diagnosis and treatment. United European Gastroenterol J.

[3] Varshney P, Kumar Kapoor V (2024). Hepato-pancreato-biliary tuberculosis: a review. Turk J Surg.

[4] Amri F, Chahi K, Mojahid A (2025). Pancreatic tuberculosis mimicking pancreatic cancer in immunocompetent patients: case series and diagnostic pathways. Radiol Case Rep.

[5] Gapizov A, Singla B, Mehta D (2025). The great mimicker: pancreatic tuberculosis masquerading as a pancreatic neoplasm. Cureus.

[6] Sharma V, Rana SS, Kumar A, Bhasin DK (2016). Pancreatic tuberculosis. J Gastroenterol Hepatol.

[7] Gu Y, Xiao M, Wan Z, Li Q (2023). Isolated pancreatic tuberculosis masquerading as malignancy in an immunocompetent host: a case report and review of the literature. J Int Med Res.

[8] Varadaraj G, Rai A, Ghana P, Maribashetti K (2024). Two cases of pancreatic tuberculosis in immunocompetent individuals presenting as diabetes mellitus: An overview of clinical features, diagnosis and management. Qatar Med J.

[9] Chaudhary P, Bhadana U, Arora MP (2015). Pancreatic tuberculosis. Indian J Surg.

[10] Saluja SS, Ray S, Pal S (2007). Hepatobiliary and pancreatic tuberculosis: a two decade experience. BMC Surg.

[11] Diaconu CC, Gheorghe G, Hortopan A (2022). Pancreatic tuberculosis-a condition that mimics pancreatic cancer. Medicina (Kaunas).

[12] Kearns MD, Tangpricha V (2014). The role of vitamin D in tuberculosis. J Clin Transl Endocrinol.

[13] Jeon CY, Murray MB (2008). Diabetes mellitus increases the risk of active tuberculosis: a systematic review of 13 observational studies. PLoS Med.

[14] Chuabio V, Bandoy J, Ong A, Te M 3rd, Maralit R (2024). Pancreatic masses clinically diagnosed as tuberculosis: Case reports. SAGE Open Med Case Rep.

[15] Liu W, Zhang B, Liu T, Jiang J, Liu Y (2024). Artificial intelligence in pancreatic image analysis: a review. Sensors (Basel).

